# Instrument completion and validation of the patient-reported apnea questionnaire (PRAQ)

**DOI:** 10.1186/s12955-018-0988-6

**Published:** 2018-08-03

**Authors:** Inger L. Abma, Maroeska Rovers, Marijke IJff, Bernard Hol, Gert P. Westert, Philip J. van der Wees

**Affiliations:** 1 Radboud Institute of Health Sciences, IQ healthcare, Radboud University Medical Center, PO box 9101, huispost 114, 6500 HB Nijmegen, the Netherlands; 2 Radboud Institute of Health Sciences, Departments for Health Evidence and Operating Rooms, Radboud University Medical Center, Nijmegen, The Netherlands; 3 ApneuVereniging, Doorn, The Netherlands; 4 Albert Schweitzer Ziekenhuis, Sleep Center, Dordrecht, The Netherlands

**Keywords:** Obstructive sleep apnea, Instrument development, Patient-reported outcome measure, Psychometrics

## Abstract

**Background:**

We previously developed the preliminary version of the Patient-Reported Apnea Questionnaire (PRAQ), a questionnaire measuring health-related quality of life in patients with (suspected) obstructive sleep apnea (OSA). This questionnaire was developed for clinical practice, where it can potentially serve two goals: use on an individual patient level to improve patient care, and use on an aggregate level to measure outcomes for quality improvement at a sleep center. In this study we aim to finalize the PRAQ, make a subselection of items and domains specifically for outcome measurement, and assess the validity, reliability and responsiveness of the PRAQ.

**Methods:**

Patients with suspected OSA were included and asked to complete the PRAQ and additional questionnaires one or more times. The collected data was used to perform the final item selection for clinical practice and for outcome measurement, create the domains for outcome measurement, and assess the measurement properties internal consistency, test-retest reliability, convergent validity and responsiveness.

**Results:**

180 patients were included in the study. The final version of the PRAQ for use in clinical practice contains 40 items and 10 domains. A subselection of 33 items in 5 domains was selected for optimal outcome measurement with the PRAQ. The results for the outcome measurement domains were: Cronbach’s alpha 0.88–0.95, ICC 0.81–0.88, and > 75% of hypotheses correct for convergent validity and responsiveness.

**Conclusions:**

The PRAQ shows good measurement properties in patients with (suspected) OSA.

**Electronic supplementary material:**

The online version of this article (10.1186/s12955-018-0988-6) contains supplementary material, which is available to authorized users.

## Background

Patients with obstructive sleep apnea (OSA) experience breathing stops while asleep, causing symptoms during the day such as excessive sleepiness, tiredness, and irritability. This can have a large impact on daily functioning of patients, and often affects a patient’s relationships and psychological wellbeing [1–3]. Furthermore, OSA is a known risk factor for comorbidities such as diabetes and heart failure [4–6], and is also associated with depression and anxiety [7–9]. Gaining an overview of the problems that OSA patients may experience, before, during, and in evaluating treatment, may be a challenge.

Patient-reported outcome measures (PROMs) are questionnaires for patients about symptoms or daily functioning. Most PROMs have been developed for use in clinical trials, but interest in their use in daily practice is growing [10, 11]. There, PROM scores can be used on an individual patient level to help bring patients’ problems to the forefront during consultations and to monitor treatment response, or on an aggregate level across groups of patients for quality improvement purposes [12]. Use of a PROM on an individual patient level may be especially relevant when patient symptoms are multiple and complex. We therefore believe that it would be beneficial to employ a PROM for patients with OSA in daily clinical practice.

In a recently published article, we described the item generation and preliminary item selection of a PROM for patients with (suspected) OSA: the Patient-Reported Apnea Questionnaire (PRAQ) [13]. We used the input of patients with OSA and healthcare professionals to select topics and items important for measuring quality of life for this patient group, which are also useful to discuss during an intake or follow-up consultation.

There are two ways in which the preliminary version of the PRAQ requires further development. First, the item reduction for the topic “sleepiness” has not yet taken place. During the item selection process of the PRAQ, patients indicated that the number of items on the topic of sleepiness could be reduced. Since the patients had no preference for which items to exclude, we decided to perform the final item reduction after studying the psychometric properties of the items. Second, the factor structure of the PRAQ has not yet been studied, and we wanted to find the optimal way to group (a subset of) the items of the PRAQ into domains for the purpose of outcome measurement.

Our aim is for the PRAQ to be employed in the following way: patients complete all items of the PRAQ before their consultation, the results of which can be discussed with a healthcare professional; and the aggregate outcomes of groups of patients can then be studied by making use of a subset of the completed items. This is beneficial for patients, who get feedback from clinicians on their results; for physicians, who get a quick insight into their patients’ main problems; and for sleep centers that wish to collect outcome data for quality improvement, because it ensures a steady stream of data due to integration in clinical practice.

In this article we describe the further development of the preliminary PRAQ. In addition, we aim to determine the reliability, validity and responsiveness of the PRAQ, with a focus on the domains that will be used for outcome measurement.

## Methods

### Population & method of completion of the PROMs

#### Baseline measurement

Patients referred to the sleep center of the Albert Scheitzer Hospital in Dordrecht, The Netherlands for suspected OSA received an invitation by email to complete the PRAQ and additional PROMs, 2–3 weeks before their intake consultation. They were informed that the results of the PRAQ would be discussed during their intake consultation. A reminder was sent one week later if the PROMs were not yet completed at that time. Patients who had not completed the PRAQ at home were offered the option of completing the PRAQ at the sleep center before their consultation.

#### Retest measurement

In order to assess test-retest reliability, patients who had completed the baseline measurement at home were asked to complete it again immediately before their intake consultation, on a computer in a private area of the sleep center. Only patients who had completed the retest no less than 7, and no more than 21 days after the baseline measurement were included for assessment of test-retest reliability.

#### Follow-up measurement

A common measure to express the number of (partial) breathing stops experienced while asleep is the Apnea-Hypopnea Index (AHI). We measured the responsiveness of the PRAQ in patients with an AHI ≥ 15, which indicates moderate to severe sleep apnea [14], and who were prescribed continuous positive airway pressure (CPAP) after their intake consultation. CPAP is the preferred treatment for OSA [15]. If the patients were still using CPAP at the time of the first follow-up consultation (6–8 weeks after start of CPAP), they were included for responsiveness. They were asked to complete the PRAQ and the additional PROMs immediately before their follow-up consultation at the sleep center. Ideally, responsiveness should be determined in a patient group in which CPAP therapy is successful and therefore a substantial change is expected with regard to the patient’s symptoms. CPAP therapy is generally considered successful when compliance is ≥4 h nightly [16].

A secure website was used for the completion of the PROMs. For any of the measurements, patients who were unable or unwilling to use a computer were offered the option of completing a paper copy of the PROMs.

### Final stage of PRAQ development

The development article of the preliminary PRAQ [13] shows how the initial 43 items were selected based on their relevance for clinical practice and were sorted into preliminary domains: symptoms at night (6 items), sleepiness (8 items), tiredness (3 items), daily activities (5 items), unsafe situations (2 items), memory and concentration (2 items), quality of sleep (2 items), emotions (6 items), social interactions (8 items), and health concerns (1 item) (Appendix 1). All items are scored on a 7-point Likert scale (higher scores indicate worse problems), and the average item scores in a domain form its domain score.

First, we performed item reduction on the sleepiness domain, as 8 items was deemed too much by patients. Then, we looked at how the PRAQ could be best used for outcome measurement. It is important that all items fit into a domain that is either “coherent” in terms of clinical relevance, or (preferably) in terms of covariance matrix as determined by principal component analysis (PCA). Therefore, our aim was to identify which items of the PRAQ can be grouped into domains for outcome measurement after use of the results of the PRAQ for an individual patient. We describe below how we first reduced the number of items for the domain ‘sleepiness’, and then how from the remaining items a subset of items was selected for outcome measurement.

### Item reduction of the sleepiness domain

During the development of the PRAQ, patients indicated that they felt that the number of items on the topic of sleepiness could be reduced. Because they had no preference for which items should be excluded, we took a statistical approach. We first looked for items with a high inter-item correlation (> 0.9), indicating that one of these items can be removed without a substantial loss of information [17]. As a second step, we used exploratory factor analysis to identify potential items with lower factor loadings (< 0.5), indicating that they do not cover the construct as well as the other items and are therefore more suitable for removal [17, 18].

### Creating domains for the PRAQ-outcome

Two of our preliminary domains, ‘symptoms at night’ and ‘social interactions’, we considered formative rather than reflective domains: they do not aim to measure aspects of the same latent construct, but the items are grouped together based on clinical relevance. Grouping items in this way can be considered a “clinimetric” approach, as opposed to a “psychometric” approach which uses statistical methods to determine the dimensionality of a PROM [19]. We wanted to group these items together irrespective of their covariance matrix, because for content reasons we did not consider it desirable to combine these items with any of the other (potential) domains. Therefore, we excluded them from the PCA and kept these domains as they were.

We performed a PCA with oblique rotation (because correlations between the different patient complaints were expected) on the 26 items of the other preliminary domains. Items that did not load on any domain with a factor loading of at least 0.5 or that had a factor loading of > 0.3 on more than one factor [17], were then one by one removed from the analysis, starting with those items that for content reasons did not seem to fit well with the items they were grouped with in the PCA. Additionally, since domains should ideally consist of at least three items, we used this as a requirement for the PRAQ-outcome domains [17]. The one-dimensional domains that were identified by the analysis were added to the two clinimetric domains. Together, these domains form the subset of the PRAQ that can be used for outcome measurement.

### Assessment of measurement properties

We studied the distribution of the individual items and the PRAQ domain scores at baseline to check for floor and ceiling effects (i.e. whether < 15% of the respondents achieved the highest or lowest possible scores [20]). We assessed the reliability, validity and responsiveness of the PRAQ following the taxonomy of measurement properties as constructed by the COSMIN panel [21].

We calculated the internal consistency parameter Cronbach’s alpha which should have a value between 0.70 and 0.95 [20]. We assessed test-retest reliability by calculatingthe intraclass correlation coefficient (ICC_consistency)_ for each PRAQ domain. ICC values of 0.7 are considered acceptable, but values of ≥0.8 are preferred [17]. Additionally, we calculated the standard error of measurement (SEM).

We used hypothesis testing to assess convergent validity, which involves studying the correlations of the scores of the PROM under study with the scores of other PROMs. We hypothesized on the size and direction of the (Spearman’s) correlations of the PRAQ domains with the (subscales of) PROMs with similar constructs (Appendix 2). We also hypothesized which PROMs should have a lower correlation with the PRAQ domain. Good convergent validity means that 75% of hypotheses are correct [20]. We used the following (subscales of) PROMs for convergent validity in their official Dutch translations:

- The Epworth Sleepiness Scale (ESS) [22], measuring daytime sleep propensity. For eight situations, a patient indicates the likelihood that they would fall asleep while in that situation. The measurement properties of the ESS have been studied in a sleep apnea population [23].
- The “vitality” domain of the RAND-36 [24]. The (freely available) RAND-36, which is the predecessor of the well-known SF-36, measures general quality of life in several domains. The vitality domain of the RAND-36 contains 4 items about a patient’s perceived energy level. The items are identical to the items of the vitality domain of the SF-36, and the domain’s measurement properties have been studied in a sleep apnea population in that context [23].
- The following short-forms of the Patient-Reported Outcomes Measurement Information System (PROMIS) databank [25–27]: sleep disturbance (5 items), sleep-related impairment (6 items), fatigue, satisfaction with participation in social roles, ability to participate in social roles, anger, anxiety and depression (the latter 6 all contained 4 items per short-form) [28–31]. For “sleep disturbance” and “anger” these were custom short-forms with fewer items than the standard short forms, in order to reduce the number of items that patients had to complete for this study.

To assess responsiveness, we constructed hypotheses about the change scores of PRAQ in correlation to the change scores of the same instruments that were employed for hypothesis testing in construct validity (Appendix 2).

## Results

### Population characteristics

The baseline population consisted of 180 patients with suspected OSA who completed the baseline measurement. Of these patients, 105 completed the retest between 7 and 21 days (average 14 days), and 53 patients completed the follow-up measurement after 6–8 weeks of treatment with CPAP. Characteristics of these respective (sub)populations can be found in Table 1.

**Table 1:** Baseline characteristics of the study populations

	Baseline population (*n* = 180)	Test-retest population (*n* = 105)	Population with follow-up after CPAP (*n* = 53)
Gender	31.7% female	38.1% female	25.0% female
Age (mean (SD))	50.1 (12.6)	50.4 (13.0)	55.8 (10.9)
Baseline AHI (mean (SD))	25 (23) (*n* = 160^a^)	27 (25) (*n* = 96^a^)	41 (22)
BMI (mean (SD))	28.9 (4.7)	28.3 (4.6)	30.4 (4.2)
ESS score (mean (SD))	9.9 (4.7)	9.6 (4.4)	9.8 (4.7)
ESS score ≥ 11	43%	42%	40%
Sleep study (type)	43% PG /57% PSG (n = 160*)	39%PG /61% PSG (n = 96*)	43%PG/ 57%PSG
CPAP compliance (mean (SD))	N/A	N/A	6:46 h (1:40 h)
CPAP compliance ≥4 h/night	N/A	N/A	96%
AHI with CPAP (mean(SD))	N/A	N/A	2.6 (3.4)

*AHI* Apnea-Hyponea Index, *BMI* Body Mass Index, *ESS* Epworth Sleepiness Scale, PG = polygraphy, *PSG* polysomnography

a. 20 patients with suspected OSA of the total study population did for various reasons (choose to) not undergo a sleep study to determine their AHI

### Missing data

Patients completing the online PRAQ were not allowed to leave any items open (no missings allowed). Eleven patients completed the PRAQ on paper one or more times, and in one of these completed PRAQs (for follow-up after CPAP), item 33 (Appendix 1) was missing from the domain “social interactions”. We computed the domain score for this patient as the average of the remaining items.

Seven items allowed the response item “not applicable” (see Appendix 1). Between 19 and 46% of respondents selected this response category for the respective items.

### Final stage of PRAQ development

#### Finishing the item selection of the sleepiness domain

None of the inter-item correlations in the preliminary ‘sleepiness’ domain was higher than 0.9. Principal component analysis showed that the lowest factor loading was 0.65, well above 0.5. Therefore, we took practical elimination decisions: the two items with a “not applicable” option were removed (about sleepiness while reading, and while driving a car) as well as an item about napping in the afternoon that had a different answering scale than the other items. This improves the homogeneity of the domain for patients. The final version of the PRAQ consists of 10 domains and 40 items (Appendix 1).

#### Identification and grouping of items for outcome measurement

The results of the final PCA can be found in Table 2. The items of the PRAQ domains “memory & concentration”, “sleep quality”, and “concerns about health” were removed because they did not have sufficient loading on any of the factors found in the PCA, or because the items loaded on more than one factor. The items of the PRAQ domains “tiredness” and “daily activities” loaded on a single factor rather than on two separate factors: these items were therefore combined in one domain called “energy & daily activities” for the goal of outcome measurement. The items of the PRAQ domain “unsafe situations” both loaded on one separate domain. However, since this domain contained only two items it was not added to the PRAQ-outcome.

**Table 2:** Results of the principal component analysis^ab^

Items	Factor 1	Factor 2	Factor 3
During the past 4 weeks, did you have a problem with:			
Fighting to stay awake during the day?	.290	**.727**	
Suddenly falling asleep?		**.836**	
Difficulty staying awake during a conversation?		**.636**	.222
Difficulty staying awake while watching something? (concert, movie, television)		**.858**	
Falling asleep at inappropriate times or places?		**.802**	
Feeling very tired?	**.785**		
Lacking energy?	**.856**		
Still feeling tired when you wake up in the morning?	**.790**		
In the past 4 weeks:			
How difficult was it for you to do your most important daily activity? (such as your job, studying, caring for the children, housework)	**.841**		
How often did you use all your energy on only your most important, daily activity? (such as your job, studying, caring for the children, housework)	**.940**		
How often did you use all your energy to accomplish only your most important daily activity? (such as your job, studying, caring for the children, housework)	**.825**		
How much difficulty did you have finding energy for your hobbies?	**.770**		
How difficult was it for you to get your chores done?	**.849**		
How often did you feel depressed or hopeless?	.266		**.677**
How often did you feel anxious?			**.793**
How often did you lose your temper?			**.803**
How often did you feel that you could not cope with everyday life?			**.746**
How often did you feel irritated?			**.889**
How often did you have a strong emotional reaction to everyday events?			**.875**

a. The bold font numbers indicate the highest factor loading for that item

b. Absolute factor loadings < 0.2 are not shown in the table

**Fig. 1:**
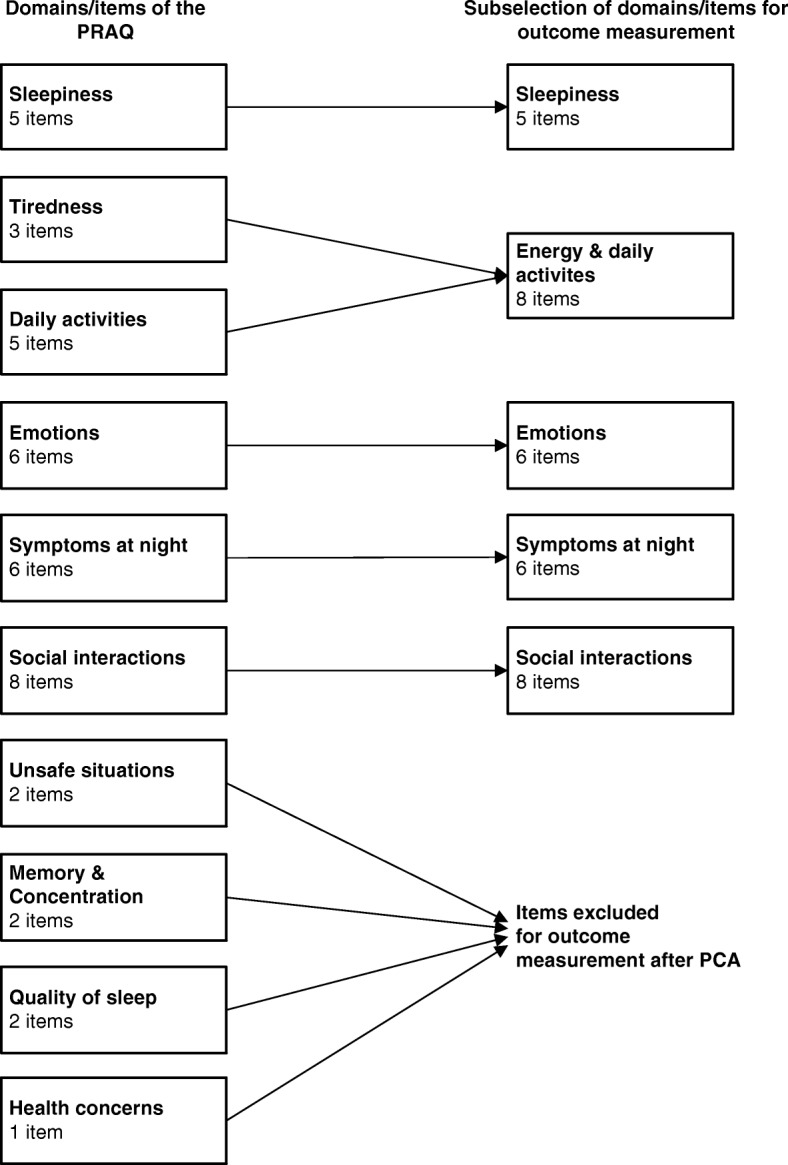
The subselection of items and domains of the PRAQ for outcome measurement

The 19 remaining items in the PCA form three one-dimensional domains: sleepiness, energy & daily activities, and emotions, which together explain 73% of the variance. The PCA showed intercorrelations of these domains of 0.36–0.57. The domains are added to the two formative domains “symptoms at night” and “social interactions”, resulting in subset of 33 items in five domains. Figure 1 illustrates how the items and domains of the PRAQ result in the subselection of PRAQ items for outcome measurement. The domains that are present in both the full 40-item PRAQ and in the 33-item outcome subset overlap to a great extent.

### Measurement properties

In this section we describe the measurement properties of the domains that are used for outcome measurement; the results for the domains of the 40-item version can be found in Additional file 1. The average baseline scores, standard deviations, and percentages of lowest and highest scores of the five outcome domains can be found in Table 3. No floor- or ceiling effects were found, except for a floor effect in the ‘sleepiness’ domain (20% of subjects scored 1–1.5). The results of the different aspects of reliability (internal consistency with Cronbach’s alpha, test-retest reliability with ICC, SEM) are also shown in Table 3. The values of Cronbach’s alpha and the ICC values are all above 0.8, indicating that these measurement properties are of good quality.

**Table 3:** PRAQ outcome domains: scores and reliability parameters (n = 180)

Domain name	Average (range 1–7)	Standard deviation	Lowest score (1–1.5)	Highest score (6.5–7)	Cronbach’s α	ICC^a^	SEM^a^
Sleepiness	3.13	1.57	20%	2.2%	0.88	0.81	0.69
Energy&daily activities	4.52	1.59	4.4%	7.8%	0.95	0.86	0.60
Emotions	2.89	1.28	13.3%	0.0%	0.92	0.85	0.50
Symptoms at night	3.48	1.27	3.9%	1.1%	- ^b^	0.88	0.44
Social Interactions	3.11	1.42	13.9%	0.6%	- ^b^	0.86	0.53

ICC = intraclass correlation efficient, SEM = standard error of measurement

a. n = 105

b. These domains are formative, and Cronbach’s α is only relevant when a domain is one-dimensional (36)

The correlations of the outcome domains with comparator instruments, which were used to determine convergent validity, are presented in Table 4. The correlations with the (somewhat) similar constructs were all within the ranges that we hypothesized (*n* = 14 hypotheses), and the correlations of selected PRAQ-domains with the dissimilar constructs were all lower than those with the similar constructs (*n* = 3 hypotheses), as expected.

**Table 4:** Correlations PRAQ outcome domains and comparator instruments ^a^ (n = 180)

	ESS	PROMIS Sleep-related impairment	RAND vitality	PROMIS fatigue	PROMIS Ability to participatie in Social Roles and Activities	PROMIS Satisfaction Social Roles	PROMIS anger/ anxiety/ depression	PROMIS sleep disturbance
Sleepiness	**0.67**	**0.60**	−.40	.52				
Energy & daily activities	0.45	**0.83**	**−.77**	**0.86**	**−0.78**	−0.60		
Emotions	0.28		−0.59	0.56	−0.60	−0.42	**0.69–0.76**	
Symptoms at night								**0.47**
Social interactions		**0.56**			**−0.52**	**−0.39**		

ESS = Epworth Sleepiness scale, PROMIS = patient-reported outcomes measurement information system

a. Correlations in bold are considered similar constructs, for which detailed hypotheses were created. The other correlations are of (somewhat) different constructs and are expected to be weaker than the bold font correlations for that PRAQ domain (for details, see Appendix 2).. Correlations for which we had no specific expectations are not shown

The absolute change scores of the PRAQ outcome domains after patients were treated with CPAP ranged from 0.76 (domain “emotions”) to 1.96 (domain “energy & daily activities”) (Appendix 3). The correlations of the change scores of the PRAQ and the change scores of the comparator instruments (Table 5) were generally in agreement with our hypotheses (*n* = 17 hypotheses). The exception was the “emotions” domain of the PRAQ, which did not correlate as strongly with the change scores of the PROMIS domains about emotions (anger, anxiety, depression; *r* = 0.26–0.43) as we had expected. When a hypothesis is not met, it is important to identify why the results are different than expected [32]. To gain more insight into these unexpected scores, we therefore ran an additional analysis on the correlation of the PRAQ scores and the PROMIS scores at the follow-up measurement, showing results of *r* = 0.62–0.71. This shows that the discrepancy lies with the change score itself and not the absolute score of the follow-up measurement.

**Table 5:** Correlations between change scores PRAQ outcome domains and comparator instruments ^a^ (n = 53)

	ESS	PROMIS Sleep-related impairment	RAND vitality	PROMIS fatigue	PROMIS Ability to participatie in Social Roles and Activities	PROMIS Satisfaction Social Roles	PROMIS anger/ anxiety/ depression	PROMIS sleep disturban-ce
Sleepiness	**.62**	**.55**	−.35	.35				
Energy&daily activities	.52	**.62**	**−.69**	**.70**	**−.74**	−.61		
Emotions	0.06	.23	−.32	.14	−.29		**.26–.43**	
Symptoms at night								**.58**
Social interactions		**.45**			**−.36**	**−.26**		

ESS = Epworth Sleepiness scale, PROMIS = Patient-Reported Outcomes Measurement Information System

a. Correlations in bold are considered similar constructs, for which detailed hypotheses were created. The other correlations are of (somewhat) different constructs and are expected to be weaker than the bold font correlations for that PRAQ-outcome domain (for details, see Appendix 2). Correlations for which we had no specific expectations are not shown

## Discussion

In this article we present the finalized Patient-Reported Apnea Questionnaire (PRAQ). The PRAQ has a unique approach with regard to the integration of use on an individual patient level and for aggregate outcome measurement: patients complete all items of the PRAQ before their consultation, the results of which can be discussed with a healthcare professional; and the aggregate outcomes of groups of patients can then be studied by making use of a subset of the completed items. The PRAQ contains all topics and items that patients and healthcare providers consider important to discuss in practice, and for this purpose includes 40 items in 10 domains. For outcome measurement, a subset of 33 items of the PRAQ was selected, divided into two formative domains (items grouped together based on what makes sense clinically) and three one-dimensional subscales. These five outcome domains generally have good measurement properties in terms of internal consistency, test-retest reliability, convergent validity and responsiveness.

PCA showed that items of the PRAQ domains “tiredness” and “daily activities” load on the same factor, which is why the items of these preliminary domains are combined into one domain for the purpose of outcome measurement. For use an individual patient level, however, we decided to keep the two domains separate. Even though we acknowledge that feeling tired (a symptom), and the extent to which daily activities can be performed normally (a consequence of that symptom), are closely related concepts, they may be relevant to discuss separately for an individual patient in clinical practice. We will test this assumption in future research, in which the PRAQ will be employed and studied empirically.

The domains that are used for outcome measurement show good responsiveness. The one exception is the domain “emotions”, the change score of which showed a much weaker correlation than expected with the change scores of PROMs with similar constructs. We hypothesize that the discrepancy between expectation and results caused by the low scores of this domain at baseline (average 2.89) and the subsequent relatively small improvement that is achieved after treatment with CPAP (average 0.76). We do not doubt the construct validity of the domain, because the comparator PROMs show the same pattern in terms of low scores and small change scores, and because the correlation of the absolute scores after treatment with CPAP shows good convergent validity. However, because the change scores are small, it is likely that measurement error plays a relatively large role in the change scores of both the PRAQ domain and the comparator instruments, reducing the accuracy of the change scores and therefore also diffusing the correlation size. This means that that in terms of outcome/quality measurement, emotional problems appear to be of less importance than the topics of the other domains and more difficult to accurately measure, because relatively few people with (suspected) OSA experience severe problems.

Surprisingly, 20% of the study population had low scores (1–1.5) on the domain ‘sleepiness’, while sleepiness is one of the main complaints of OSA. We think that this is due to a relatively high difficulty of the sleepiness items of the PRAQ (such as falling asleep during a conversation) in combination with a generally low sleepiness in this population (average ESS < 10). This reason for the low sleepiness in the population is probably twofold. First, the main complaint of some patients who were referred for suspected OSA in this study is probably (socially problematic) snoring rather than sleepiness or tiredness during the day. OSA treatment will reduce snoring and is reimbursed by healthcare insurers, making it beneficial for these patients to visit the sleep center and get an OSA diagnosis. Second, for logistical reasons some patients with suspected severe OSA were not included in the study. These patients a fast- track procedure to bypass the sleep center’s the waiting list, which meant they were in practice not always asked to join the study. This is a limitation of the study. What we can derive from the current results is that the sleepiness domain of the PRAQ seems more useful to detect cases of severe sleepiness, which definitely requires treatment, than to distinguish mild and moderate sleepiness. However, future research should take place in a more representative patient group to study how the sleepiness domain performs in this population.

The PRAQ is designed for use in clinical practice, to help focus consultations on the problems that individual patients encounter. When using a PROM for this purpose, the ICC should preferably be very high (0.9–0.95 at individual level vs. 0.8 or higher at group level for aggregate outcome measurement [17]). The ICC values of the PRAQ are lower (0.81–0.88). However, the PRAQ is meant to open the conversation about a patient’s symptoms and functioning, not to serve as a “cut-off” score. Any elevated score could therefore result in conversation about this topic, and we believe that the PRAQ can serve its purpose despite the slightly lower ICCs.

### Methodological considerations

The domains for outcome measurement were created with a combination of the “clinimetric” approach, in which items are grouped together based on clinical relevance; and the “psychometric” approach, which groups items together based on PCA [33–35]. The combination of these two approaches is uncommon. We believe that scores of psychometric domains, with a clear one-dimensional construct, are more meaningful than formative domains because they have a clear interpretation. However, this approach is not always feasible when items have been selected to be part of a quality-of-life or symptoms questionnaire based on their deemed importance by the target population [36]. Items which cover symptoms of the same disease or treatment will often share covariance and thus appear to be covering the same latent construct, even when looking at the content of the items this makes no apparent sense (e.g. lack of appetite and decreased sexual interest in patients undergoing cancer treatment [36]). Therefore, we considered the best approach grouping together the different symptoms patients experience at night, as well as the variety of different ways in which sleepiness, tiredness and emotions might influence a patients’ social life, without subjecting them to PCA.

**Fig. 2:**
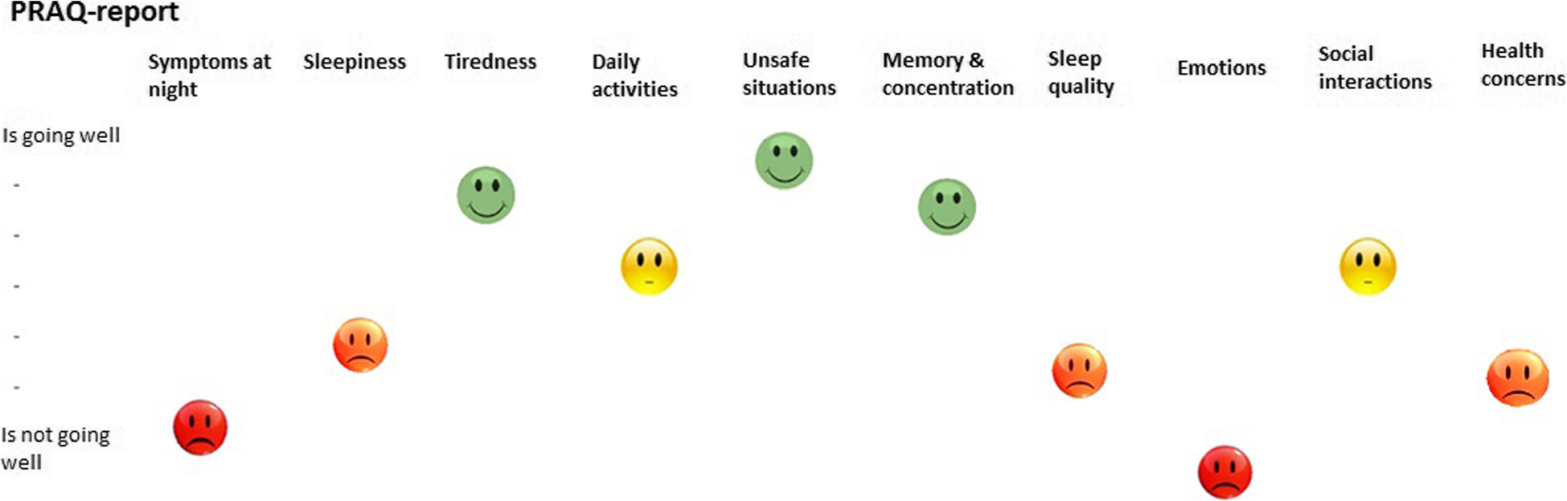
The first page of the PRAQ-report

To aid the use of the PRAQ in clinical practice, we developed a patient-friendly digital report (the PRAQ-report) together with patients and healthcare professionals (Fig. 2). When using the PRAQ in clinical practice, it can be useful to look at individual item scores as well as the domain scores, especially in the formative domains in which item scores will generally differ more from each other. Therefore, both domain and individual item scores are shown in the report.

## Conclusions

In conclusion, we have shown that the PRAQ-practice and PRAQ-outcome generally have acceptable measurement properties and appear to be suitable PROMs for their respective purposes. However, further validation research is needed in patients who suffer from higher levels of sleepiness, to study the validity of the sleepiness domain. The applicability of a PROM for use in clinical practice and for measuring outcomes on aggregate level, may be of great importance for the further implementation of PROMs in healthcare.

### Additional file

Additional file 1: Domain and reliability scores of the PRAQ clinical practice domains. (DOCX 17 kb)

## Appendix 1

**Table 6:** The Patient-Reported Apnea Questionnaire (PRAQ)

**Symptoms at night**	
During the past 4 weeks, did you have a problem with: 1. Snoring loudly? 2. Waking up frequently to urinate? 3. Waking up at night with the feeling that you are choking? 4. A feeling that you are sleeping restlessly? 5. Having a dry or painful mouth when you wake up? 6. Waking up in the morning with a headache?	
**Sleepiness**	
During the past 4 weeks, did you have a problem with: 7. Fighting to stay awake during the day? 8. Suddenly falling asleep? 9. Difficulty staying awake during a conversation? 10. Difficulty staying awake while watching something? (concert, movie, television) 11. Falling asleep at inappropriate times or places? Difficulty staying awake while reading?^a,b^ Fighting to stay awake when you are driving?^a,b^ Did you feel like you needed to take a nap in the afternoon?^a^	
**Tiredness**	
During the past 4 weeks, did you have a problem with: 12. Feeling very tired? 13. Lacking energy? 14. Still feeling tired when you wake up in the morning?	
**Daily activities**	
During the past 4 weeks: 15. How difficult was it for you to do your most important daily activity? (such as your job, studying, caring for the children, housework) 16. How often did you use all your energy to accomplish only your most important daily activity? (such as your job, studying, caring for the children, housework) 17. Did you feel you have a decreased performance with regard to your most important daily activity? (such as your job, studying, caring for the children, housework) 18. How much difficulty did you have finding energy for your hobbies? 19. How difficult was it for you to get your chores done?	
**Unsafe situations**	
During the past 4 weeks: 20. Did you have problems while driving a car due to sleepiness?^b^ 21. Were you concerned about your safety or that of others due to your sleepiness? (for example in traffic, or when operating machinery)	
**Memory and concentration**	
During the past 4 weeks: 22. Were you sometimes forgetful? 23. Did you sometimes have difficulty concentrating?	
**Quality of sleep**	
During the past 4 weeks, did you have a problem with: 24. Falling asleep when you go to bed at night? 25. Getting back to sleep after you woke up at night?	
**Emotions**	
During the past 4 weeks: 26. How often did you feel depressed or hopeless? 27. How often did you feel anxious? 28. How often did you lose your temper? 29. How often did you feel that you could not cope with everyday life? 30. How often did you feel irritated? 31. How often did you have a strong emotional reaction to everyday events?	
**Social interactions**	
During the past 4 weeks: 32. Did you sometimes feel upset because others were disturbed by your snoring? 33. Was it a problem for you that you sometimes had no energy or no desire to do things with your family or your friends? 34. Did you feel guilty towards your family or friends? 35. Did you feel upset because you argued frequently? 36. Did you sometimes experience problems in the relationship with your partner?^b^ 37. Did you feel upset because you could (maybe) not sleep in the same room as your partner?^b^ 38. Did you sometimes think up excuses because you were tired or sleepy? 39. Did you have a problem with unsatisfying and/or too little sexual activity? (by yourself or with another)^b^	
**Health concerns**	
40. Were you concerned about other conditions that may be related to sleep apnea? (such as diabetes, high blood pressure, cardiovascular disease, being overweight)	

a. The shaded items of the “sleepiness” domain were removed from this domain in the final version of the PRAQ

b. These items had an additional response option “not applicable” or (for item 39) “no answer”

## Appendix 2

**Table 7:** Hypotheses for convergent validity and responsiveness

**PRAQ domain**	**Comparison instrument**	**Expected correlation strength (direction)**	**Explanation** (hypothesis nr between brackets)
Sleepiness	ESS	0.5–0.8 (+)	The ESS asks about current daytime sleep propensity, while the PRAQ domain asks to look back on the past month and indicate how much of a *problem* sleepiness or falling asleep was. The domains do not cover the exact same construct, but are relatively similar. We expect a moderately strong correlation (h1). “Sleep-related impairment” is a domain with questions covering both sleepiness and tiredness. We expect a moderate to strong correlation with the PRAQ domain “sleepiness” (h2) because of the overlapping items on sleepiness, but also because the concept of sleepiness itself correlates with the concept of tiredness and how tiredness affects daily activities in our study population (correlation strength 0.57 as found in our principal component analysis). *Dissimilar constructs* We expect the correlations with the PROMIS fatigue and RAND-36 vitality domains to be lower than the correlations with the similar constructs of the PROMs mentioned above (h3).
	PROMIS: Sleep-related impairment	0.5–0.8 (+)	
	*Different constructs*		
	PROMIS fatigue (+) RAND-36 vitality (−)		
Energy & daily activities	PROMIS: Sleep-related impairment	0.6–0.9 (+)	“Sleep-related impairment” is a domain with questions covering both sleepiness and tiredness, in the context of daily activities. Therefore we expect a strong correlation with the PRAQ domain (h1). The RAND-36 vitality domain and the PROMIS fatigue both contain questions about tiredness. We expect both to have a strong correlation with the PRAQ domain (h2 + 3). We expect a somewhat stronger (h4) correlation with the PROMIS fatigue domain because this domain is focused on to what extent one feels tired (similar to the PRAQ), while the RAND focuses on *how often* one feels tired. The PROMIS domain “ability to participate in social roles and activities” asks question about to what extent someone is able to do their usual daily or social activities. We expect a strong correlation with the PRAQ domain because the constructs are similar (h5). *Dissimilar constructs* We expect the correlations with the ESS and the “PROMIS satisfaction with social roles and activities” domain to be lower than the correlations with the similar constructs of the PROMs mentioned above (h6).
	RAND-36 vitality	0.6–0.9 (−)	
	PROMIS Fatigue	0.6–0.9 (+)	
	PROMIS ability to participate in social roles and activities	0.6–0.9 (−)	
	*Different constructs*		
	PROMIS satisfaction with social roles and activities (−) ESS (+)		
Emotions	PROMIS anger	0.6–0.9 (+)	The three PROMIS domains contain items asking about *how often* certain emotions are felt, which is the same approach as the PRAQ-domain. The PRAQ-domain also contains all three of these types of emotions. Therefore we expect a strong correlation with all three of these domains (h1–3). *Dissimilar constructs* We expect the correlations of the PRAQ “emotions” domain with the ESS, RAND-36, and the PROMIS domains ability to participate in social roles and activities and “satisfaction with social roles and activities” to be lower than the correlations with the similar constructs of the PROMs mentioned above (h4).
	PROMIS anxiety	0.6–0.9 (+)	
	PROMIS depression	0.6–0.9 (+)	
	*Different constructs*		
	ESS (+) RAND-36 vitality (−) PROMIS ability to participate in social roles and activities (−) PROMIS satisfaction with social roles and activities (−)		
Symptoms at night	PROMIS sleep disturbance	0.2–0.6 (+)	The most similar domain that we included for the PRAQ domain “symptoms at night” is the PROMIS “sleep disturbance” domain. This domain contains items about whether patients are sleeping well. Even though a majority of the items in the PRAQ domain “symptoms at night” will affect the quality of sleep, the content of the two domains is very different. We therefore will not make a very precise hypothesis, and expect at least a low to moderate correlation (h1). Dissimilar constructs are not included for this domain. Due to the domain’s clinimetric nature it covers several topics that are related to severity of OSA and that might therefore have unpredictable correlations with other domains.
Social interactions	PROMIS: Sleep-related impairment	0.2–0.6 (+)	The “social interactions” domain of the PRAQ contains a collection of items about different social problems that apnea patients might experience due to their snoring, sleepiness, tiredness, or emotions. Because this domain does not clearly cover one single construct, we expect it to have no more than low to moderate correlations with any of the comparator PROMIS domains (h1–3). Dissimilar constructs are not included for this domain. Due to the domain’s clinimetric nature it covers several topics that are related to severity of OSA and that might therefore have unpredictable correlations with other domains.
	PROMIS ability to participate in social roles and activities	0.2–0.6 (−)	
	PROMIS satisfaction with social roles and activities	0.2–0.6 (−)	

## Appendix 3

**Table 8:** Change scores of the PRAQ outcome domains after treatment with CPAP (n = 53)

Domain name	Baseline score	Average score after treatment	Average change score ^a,b^
Sleepiness	3.27	1.56	1.70
Energy & daily activities	4.66	2.70	1.96
Emotions	2.76	2.00	0.76
Symptoms at night	3.45	1.82	1.63
Social Interactions	2.94	1.79	1.15

a. A positive change score stands for a reduction in symptoms

b. All change scores are significant, *p*-value < 0.00

## Abbreviations

AHI: apnea-hypopnea index
CPAP: continuous positive airway pressure
ESS: Epworth sleepiness scale
ICC: intraclass correlation coefficient
OSA: obstructive sleep apnea
PCA: principal component analysis
PRAQ: patient-reported apnea questionnaire
PROM: patient-reported outcome measure
PROMIS: patient-reported outcomes measurement information system
SEM: standard error of measurement
