# Supplementary material for: Instrument completion and validation of the patient-reported apnea questionnaire (PRAQ)
Source: Health Qual Life Outcomes. 2018 Aug 3;16:158. doi: 10.1186/s12955-018-0988-6 (PMC6090652; doi:10.1186/s12955-018-0988-6)
Supplement: Supplementary file 1 — Domain and reliability scores of the PRAQ clinical practice domains. (DOCX 17 kb) [file 12955_2018_988_MOESM1_ESM.docx]

**Additional file 1 – Domain and reliability scores of the PRAQ clinical practice domains**

**Scores** **of the PRAQ clinical practice domains**^a^

| **Domains** | **Average**  **(range 1-7)** | **Standard deviation** | **Lowest score**  **(1-1.5)** | **Highest score (6.5-7)** |
| --- | --- | --- | --- | --- |
| Sleepiness | 3.13 | 1.57 | 20% | 2.2% |
| Tiredness | 4.85 | 1.71 | 4.4% | 20.0% |
| Daily activities | 4.33 | 1.64 | 5.0% | 8.9% |
| Emotions | 2.89 | 1.28 | 13.3% | 0.0% |
| Symptoms at night | 3.48 | 1.27 | 3.9% | 1.1% |
| Social Interactions | 3.11 | 1.42 | 13.9% | 0.6% |
| Memory&concentration | 3.85 | 1.34 | 4.4% | 1.7% |
| Unsafe situations | 2.13 | 1.56 | 57% | 1.1% |
| Sleep quality | 2.71 | 1.71 | 42.8% | 6.7% |
| Health concerns | 3.34 | 2.00 | 25.6% | 8.9% |

a. The clinical practice domains partially overlap with the outcome domains of the PRAQ, as shown in Figure 1 in the body of the article.

**Reliability scores of the PRAQ clinical practice domains**^a^

| **Domains** | **Cronbach’s α** | **ICC** | **SEM** |
| --- | --- | --- | --- |
| Sleepiness | 0.88 | 0.81 | 0.69 |
| Tiredness | 0.93 | 0.86 | 0.64 |
| Daily activities | 0.94 | 0.83 | 0.68 |
| Emotions | 0.92 | 0.85 | 0.50 |
| Symptoms at night | - ^b^ | 0.88 | 0.44 |
| Social Interactions | - ^b^ | 0.86 | 0.53 |
| Memory&concentration | 0.83 | 0.86 | 0.50 |
| Unsafe situations | 0.87 | 0.87 | 0.56 |
| Sleep quality | 0.72 | 0.83 | 0.71 |
| Health concerns^c^ | - | - | - |

SEM = standard error of measurement

a. The clinical practice domains partially overlap with the outcome domains of the PRAQ, as shown in Figure 1 in the body of the article.

b. These domains are formative, and Cronbach’s α is only relevant when a domain is one-dimensional (1).

c. This domain contains only one item, meaning these measurement properties cannot be calculated.

**References**

1. Streiner DL. Being inconsistent about consistency: when coefficient alpha does and doesn't matter. J Pers Assess. 2003; 80: 217-22.
